# Analysis of amplicon-based NGS data from neurological disease gene panels: a new method for allele drop-out management

**DOI:** 10.1186/s12859-016-1189-0

**Published:** 2016-11-08

**Authors:** Susanna Zucca, Margherita Villaraggia, Stella Gagliardi, Gaetano Salvatore Grieco, Marialuisa Valente, Cristina Cereda, Paolo Magni

**Affiliations:** 1 Department of Electrical, Computer and Biomedical engineering, University of Pavia, Pavia, 27100 Italy; 2 Center of Genomics and post-Genomics, IRCCS National Institute of Neurology Foundation “C. Mondino”, Pavia, 27100 Italy

**Keywords:** Next-generation sequencing, Amplicon-based sequencing, Allele drop-out, Bioinformatic pipeline, Primer trimming

## Abstract

**Background:**

Amplicon-based targeted resequencing is a commonly adopted solution for next-generation sequencing applications focused on specific genomic regions. The reliability of such approaches rests on the high specificity and deep coverage, although sequencing artifacts attributable to PCR-like amplification can be encountered. Between these artifacts, allele drop-out, which is the preferential amplification of one allele, causes an artificial increase in homozygosity when heterozygous mutations fall on a primer pairing region.

Here, a procedure to manage such artifacts, based on a pipeline composed of two steps of alignment and variant calling, is proposed. This methodology has been compared to the Illumina Custom Amplicon workflow, available on Illumina MiSeq, on the analysis of data obtained with four newly designed TruSeq Custom Amplicon gene panels.

**Results:**

Four gene panels, specific for Parkinson disease, for Intracerebral Hemorrhage Diseases (COL4A1 and COL4A2 genes) and for Familial Hemiplegic Migraine (CACNA1A and ATP1A2 genes) were designed.

A total of 119 samples were re-sequenced with Illumina MiSeq sequencer and panel characterization in terms of coverage, number of variants found and allele drop-out potential impact has been carried out. Results show that 14 % of identified variants is potentially affected by allele drop-out artifacts and that both the Custom Amplicon workflow and the procedure proposed here could correctly identify them.

Furthermore, a more complex configuration in presence of two mutations was simulated *in silico*. In this configuration, our proposed methodology outperforms Custom Amplicon workflow, being able to correctly identify two mutations in all the studied configurations.

**Conclusions:**

Allele drop-out plays a crucial role in amplicon-based targeted re-sequencing and specific procedures in data analysis of amplicon data should be adopted. Although a consensus has been established in the elimination of primer sequences from aligned data (e.g., via primer sequence trimming or soft clipping), more complex configurations need to be managed in order to increase the retrieved information from available data. Our method shows how to manage one of these complex configurations, when two mutations occur.

**Electronic supplementary material:**

The online version of this article (doi:10.1186/s12859-016-1189-0) contains supplementary material, which is available to authorized users.

## Background

In the last 30 years, Sanger sequencing has been the gold standard technique in molecular diagnostics. Recent years have witnessed the advent of Next Generation Sequencing (NGS) technologies that have greatly improved sequencing capability, while dramatically decreasing the cost per sequenced base. NGS techniques generate high-throughput genomic data and specific analysis procedures (called bioinformatic pipelines) are currently developed to extract the information of interest from the extremely large amount of raw data generated as output from NGS experiments. While whole-genome and whole-exome sequencing experiments are exploited to investigate the entire genetic heritage of an individual, targeted re-sequencing applications have been introduced for those investigations where only small, user-defined portions of the genome need to be sequenced. This last approach is widely employed to study single- or multi-gene disorders [1].

Amplicon-based applications for targeted re-sequencing are a commonly adopted solution [2, 3]. These approaches are based on the design of synthetic oligonucleotides (or probes), with complementary sequence to the flanking regions of the target DNA to be sequenced.

Commercial gene panels are available to investigate widely studied diseases (e.g.: Illumina TruSeq Amplicon Cancer Panel, Illumina TruSight Myeloid Amplicon Panel), while customized gene panels can be designed to meet the specific requirements (e.g.: Illumina TruSeq Custom Amplicon, Life Technology’s AmpliSeq). Multi-gene custom panels for neurological diseases are today currently employed in both research and diagnostics [2, 4, 5].

Amplicon-based sequencing approaches are characterized by high specificity and deep coverage [1] and have been successfully employed both with good-quality DNA sources such as blood or frozen tissues and with more challenging samples extracted from formalin-fixed and paraffin-embedded tissues [6]. Since amplicon-based sequencing is still based on PCR amplification, some of the artifacts that can be encountered in traditional Sanger sequencing are still present, such as nucleotide misincorporation by polymerase, chimera formation (amplicons containing motifs from different alleles) and allelic drop-out (ADO, preferential amplification of one allele, causing an artificial increase in homozygosity values) [7–10].

Several efforts to manage such artifacts have been attempted, including the progressive development of gold standard rules for PCR, based on the use of independent amplification reactions, the reduction of PCR cycle number, the increase of elongation time and the addition of a reconditioning step [11, 12]. Other approaches are based on the modification of experimental design by introducing additional redundant overlapping amplicons to over-cover the target regions [2].

Further methodologies include the use of replicated amplicons and of a specific workflow to classify each amplicon as a putative allele or an artifact [7]. Advances in the bioinformatics field led to the creation and the development of algorithms to manage such artifacts during the analysis (e.g.: AmpliVar [13], TSSV [14] and Mutascope [15]). AmpliVar is based on the reduction of the number of input reads to be aligned to a reference genome by grouping for primer sequence in a key-value structure, where each group is analyzed independently [13]. TSSV is a tool specifically designed to profile all allelic variants present in targeted locus, able to detect and characterize complex allelic variants, such as short tandem repeats [14]. Mutascope is a software dedicated to the detection of mutations at low-allelic fraction from amplicon sequencing of matched tumor-normal sample pairs, based on variant classification as somatic or germline via a Fisher exact test [15]. New bioinformatic pipelines, based on primer trimming and perfected variant calling have also been developed and tested on synthetic amplicon datasets [16]. Also, manufacturer’s proprietary software for the analysis of amplicon-based data is available, like Ilumina MiSeq Reporter Custom Amplicon workflow (Illumina, Inc., San Diego, CA), based on primer sequence soft-clipping and on the alignment of each read with the expected amplicon, thus obtaining a fast and reliable variant identification procedure. AmpliVar and Mutascope performances were compared to Illumina workflow on five separate amplicon assays [13]: AmpliVar sensitivity was higher than Mutascope and variant identification was in full accordance with Illumina workflow. It is worth noting that none of the previously described tools is designed to manage ADO artifacts, with the exception of Illumina workflow, via primer sequence soft-clipping.

ADO entity is tremendously variable, depending on the type and on the position of the primer-sequence mismatch. Single nucleotide mismatches occurring at the 3’ terminus of a primer can dramatically affect amplification efficiency (with a yield reduction up to 100-fold), depending on mismatch position (e.g.: last four bases are the most affecting amplification efficiency) and on mismatch nature (e.g.: A:G, G:A and C:C are the worst combinations) [17–20]. Widely used, online available tools for primer design show that a single nucleotide mismatch can lower primer melting temperature up to 18 °C [21], with serious impacts on process efficiency. Nucleotide insertion/deletions have an even more disruptive effect.

In this paper, we have considered the exemplificative configuration of a single nucleotide mismatch and hypothesized the worst-case configuration of a yield reduction up to 100-fold, but drawn conclusions can be extended to more favorable cases with mitigated yield reduction and to more general cases with insertions or deletions falling on primer-matching sequence.

**Fig. 1:**
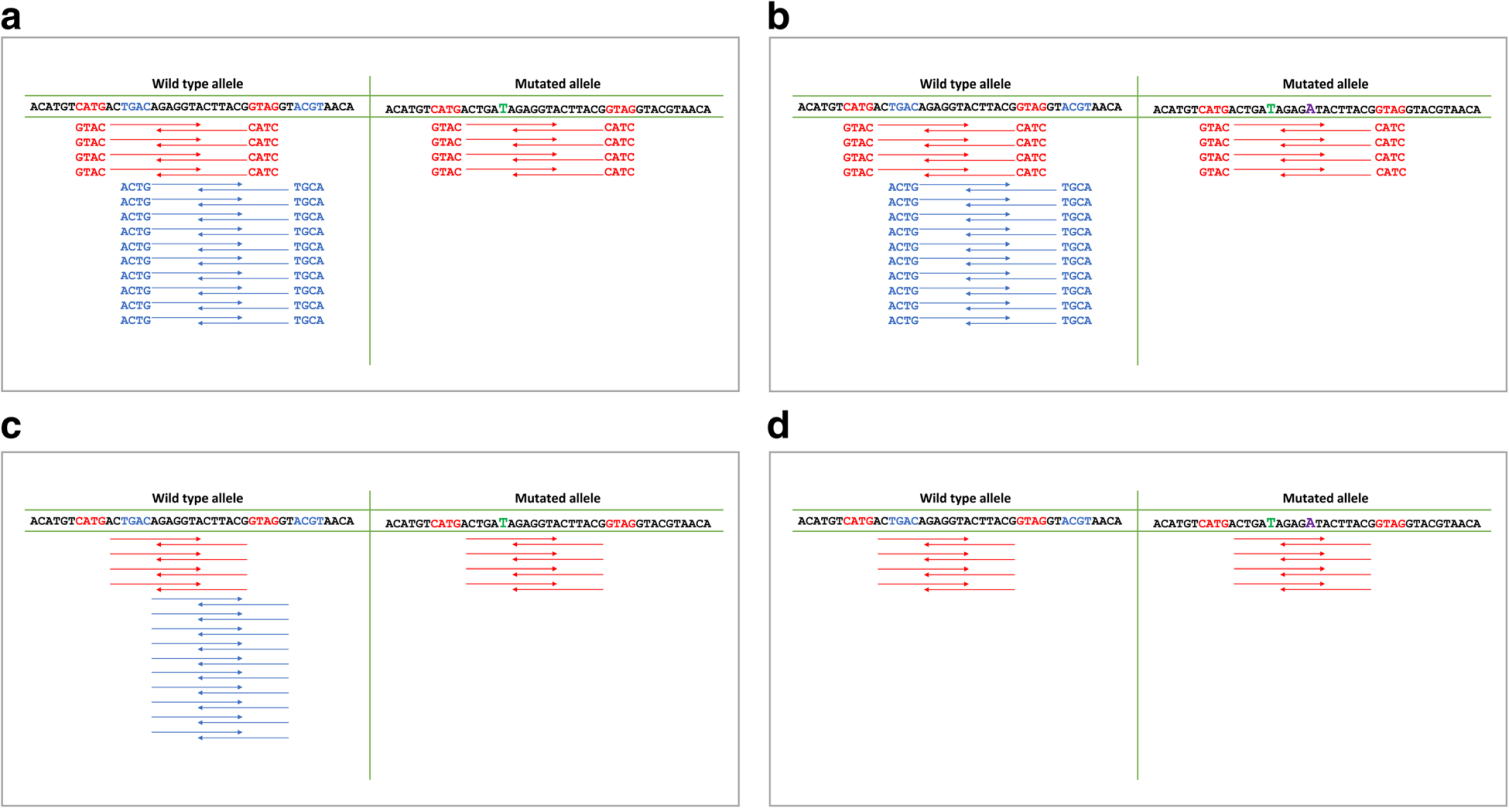
Allele Drop-Out related artifacts in variant identification. **a** One mutated (C > T, in green) and one wild type allele are shown. Only amplicons originated from primers pairing a non mutated region (in red) are randomly generated by both alleles, while primers pairing the mutated region (in blue) preferentially amplify the wild type allele. **b** In this configuration, a second mutation (G > A, in purple) is also present on the mutated allele. This mutation is masked by ADO effects, since the mutated allele is never amplified by blue primers. **c** Primer trimming (i.e., the removal of primer sequences from reads) restores the balance of aligned bases in the mutated position. **d** Primer trimming in this context is not sufficient to restore a balanced number of reads in the position relative to the second mutation. In order to do this, one possible approach is the removal of blue reads, generated by the amplicon affected by ADO-artifacts

An exemplificative configuration in which ADO-related artifacts can affect variant discovery is reported in Fig. 1a. Here, two alleles are represented, one of them containing a single nucleotide variant (C > T, green). The wild type allele (on the left) perfectly pairs with both the red and blue primer couples, generating both blue and red amplicons, not containing the mutation. The mutated allele (on the right) perfectly matches the red primer sequence only, thus generating the red amplicon, containing the mutated sequence. The blue amplicon is rarely generated by the mutated allele, since, we suppose, imperfect sequence matching between primer and sequence biases amplicon formation towards wild-type allele. During variant calling step, the reads containing the mutation account for a fraction that is often neglected by variant callers (<25 % of total reads), thus resulting in a false homozygosity. An even more complex configuration is represented in Fig. 1b, where an additional mutation (G > A, purple) is present. This mutation is present in heterozygous state, on the same allele containing the mutation illustrated above (C > T, green). Although not falling on a primer matching sequence, this second mutation is hidden and variant calling issues are the same as illustrated before.

**Fig. 2:**
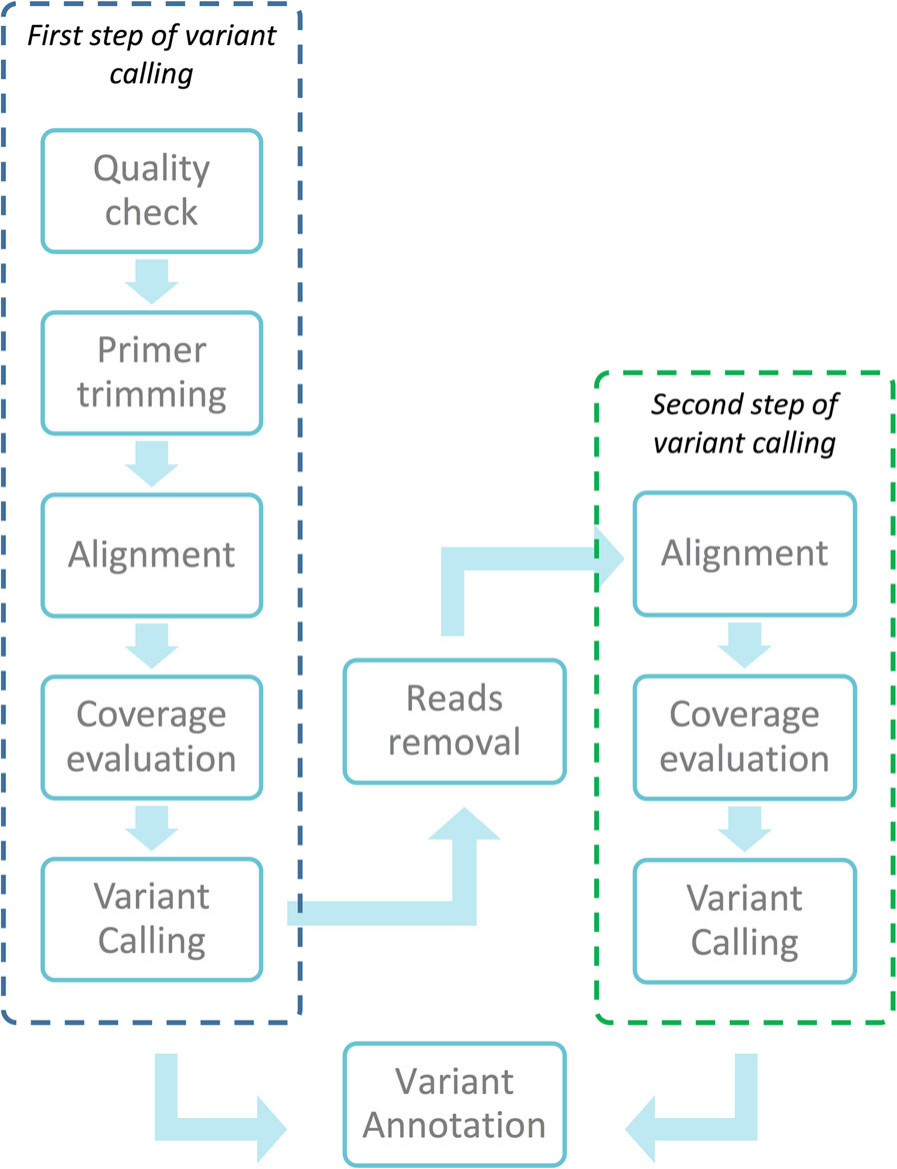
Schematic representation of trimming algorithm, based on two separate steps of alignment and variant calling. The first step is characterized by primer sequence trimming, the second step by the removal of reads generated by primer pairs that pair in a mutated region, with the mutation identified during the first step of variant calling. Variants obtained in the two different steps are merged, annotated, and provided as output from this pipeline

We designed a methodology to prevent ADO artifacts, based on a first step of alignment and variant calling after primer sequence trimming (Fig. 1c) and on a second step, after removal of reads generated from primers matching a sequence containing a mutation identified at the first step (Fig. 1d). This methodology exploits the redundancy of amplicon coverage on target regions to maximize the retrieved information from available data. The procedure is summarized in Fig. 2.

With this methodology, we have analyzed 119 samples, obtained from four newly designed Illumina TruSeq Custom Amplicon gene panels related to neurological diseases. Results have been compared with the MiSeq Reporter Custom Amplicon workflow output and a subset of putative representative mutations identified via this procedure, considered by genetists to be clinically relevant for the studied phenotype, has been validated via Sanger sequencing. A synthetic dataset has also been constructed to allow the comparisons of these methodologies for variant calling when two mutations (single nucleotide mismatch or insertion) are present on the same allele. A synthetic dataset corresponding to a representative configuration was also analyzed with AmpliVar tool [13].

## Methods

### TruSeq Custom Amplicon gene panels and sequencing experiments

Illumina TruSeq Custom Amplicon Kit was used to capture all exons, intron–exon boundaries, 5’- and 3’-UTR sequences and 10-bp flanking sequences of target genes (RefSeq database, hg19 assembly).

Four different gene panels, related to neurological diseases, were *de-novo* designed, as shown in Table 1.

**Table 1:** The four gene panels designed and adopted in this study

	Genes	Number of amplicons	Cumulative target (bps)	Number of samples
Parkinson panel	GBA, ATP13A2, PARK7, PINK1, EIF4G1, UCHL1, SNCA, PARK2, LRRK2, VPS35	368	76,146	54
COL4 panel	COL4A1, COL4A2	157	31,842	23
CACNA1A panel	CACNA1A	83	16,837	27
ATP1A2 panel	ATP1A2	57	10,244	15

For each panel, the genes involved are reported, together with the total number of amplicons per panel and the dimension of the target regions in base pairs. For each panel, the number of sequenced samples is reported

Parkinson panel is composed by ten known causative genes for Parkinson disease, both for the autosomal dominant and recessive forms [22–24].

Both CACNA1A and ATP1A2 panels are monogenic. Mutations in CACNA1A gene determine two allelic disorders with a dominant-autosomic transmission: Spinocerebellar Ataxia 6 and Episodic Ataxia 2 [25]. Furthermore, mutations have been described in patients with alternating hemiplegia and recurrent ischemic stroke [26]. Mutations in ATP1A2 are reported in case of alternating hemiplegia [27]. Both genes are causative for familial and sporadic hemiplegic migraine [28, 29].

COL4 panel contains COL4A1 and COL4A2 genes, the mutations of which contribute to a broad spectrum of disorders, including myopathy, glaucoma and hemorrhagic stroke [30–32].

For the four studied gene panels, probes were designed using DesignStudio (http://designstudio.illumina.com/) and amplicon length averaged 250 base pairs (2×150 base pairs reads length in paired-end mode). Amplicon number varied from 57 to 368 (see Table 1).

For this work, 119 patients with suspected diagnosis for the studied diseases have been recruited at “C. Mondino” National Institute of Neurology Foundation (Pavia, Italy) and from other clinics in Italy.

Peripheral blood samples were collected after obtaining written informed consent (approved by the Ethics Committee) from all the participants and genomic DNA was purified by automatic extraction (Maxwell® 16 Blood DNA – Promega).

The TruSeq Custom Amplicon sequencing assay was performed according to manufacturer’s protocol (Illumina, Inc., San Diego, CA). All DNA samples were diluted to the same initial concentration (25 ng/μl). In order to artificially increase the genetic diversity, 10 % DNA from phage PhiX was added to the library of genomic DNA before loading on the flow-cell [3].

Sample normalization has been performed according to Illumina manufacturer protocol to get a concentration of 10 nM per sample. PAL (Pooled Amplicon Library) preparation has been performed according to manufacturer’s protocol. 6 μl of PAL were diluted in 600 μl of DAL (Diluted Amplicon Library) and then loaded on the flow cell.

Runs were performed on Illumina MiSeq sequencer with V2 flow cell. Reagent cartridges were purchased from Illumina (MS*300 V2 series).

Six sequencing experiments have been carried out, with an average sample number of 34 samples per run (min: 23, max: 63). Five experiments have involved a single gene panel, while in one experiment samples analyzed with CACNA1A and ATP1A2 panels have been pooled. In this case, in the final pool realization, a normalization on the amplicon number was performed in order to have the same expected average coverage for all the samples, although CACNA1A and ATP1A2 panels already have similar dimension.

When specified, candidate genes were amplified by PCR using primers located in adjacent intronic regions from genomic DNA. The amplicons were screened for sequence variations by direct sequencing using the Big-Dye Terminator v3.1 sequencing kit (Applied Biosystems, Milan, Italy) and ABI 3130 Genetic Analyzer (Applied Biosystems, Milan, Italy). The alignment to reference sequence has been performed using Sequencher 4.8 software.

### Bioinformatic data analysis

#### Primary analysis

Data collected from NGS experiments were analyzed in order to identify single nucleotide variants and small insertions/deletions.

The first steps of bioinformatic analysis (including base calling and demultiplexing) have been performed using MiSeq provided software (Real Time Analysis RTA v.1.18.54 and Casava v.1.8.2, Illumina, Inc., San Diego, CA). FastQ files provided for each sample, containing mate paired-end reads after demultiplexing and adapter removal, were used as input for two different pipelines.

#### MiSeq pipeline

First, FastQ files were processed with MiSeq Reporter v2.0.26 using the Custom Amplicon workflow (hereinafter called “MiSeq pipeline”). This analytical method requires as input both FastQ files with forward and reverse reads and a “Manifest file” containing information about the sequences of primer pairs, the expected sequence of the amplicons and the coordinates relative to the reference genome (Homo sapiens, hg19, build 37.2). As output, a VCF file is generated, containing the list of the identified mutations. Briefly, each read pair is separately processed to individuate the corresponding primer pair (allowing one mismatch) and then aligned to the expected amplicon sequence (primers excluded) via banded Smith-Waterman algorithm, accepting gaps up to one third of its length (http://support.illumina.com/content/dam/illumina-support/documents/documentation/chemistry_documentation/samplepreps_truseq/truseqcustomamplicon/truseq-custom-amplicon-15-reference-guide-15027983-02.pdf). The alignment BAM file thus obtained is then provided as input to GATK variant caller (Genome Analysis ToolKit, v1.6 [33]) that generates a VCF file for each sample.

#### Trimming pipeline

The second bioinformatic pipeline (hereinafter called “trimming pipeline”) implements the algorithm shown in Fig. 2 and receives as input both FastQ files (forward and reverse reads) and Manifest file. First, a quality control check is implemented with FastQC tool [34] and only samples with sufficient number of reads and base quality are considered. These thresholds were *a posteriori* empirically determined based on the 20 % of samples for each panel showing the smaller number of uncovered regions and considering the average quality and number of reads per sample as a reference. Samples with average reads quality lower than 30 % of the reference or total number of reads lower than 10 % of the reference were excluded. Then, a primer sequence trimming step is performed (Fig. 1c) via ad-hoc developed Perl scripts (*generate_primer_list.pl* and *trimming.pl*). Here, forward and reverse oligonucleotide sequences (called Upstream and Downstream Locus Specific Oligos, ULSO and DLSO, respectively) are extracted from manifest file and used to match read pairs. Only read pairs matching a primer pair, accepting one mismatch per read and no gaps, are maintained and used for further analysis. More in detail, the first read mate is aligned against the forward primer sequence. If the primer sequence entirely matches the first bases of the reads, allowing one mismatch and no gaps, it is trimmed off from the read. If no primer sequence is identified, the entire read pair is discarded. The mate read is also aligned against the reverse primer sequence. In case of sequence matching (with the same criteria admitted above), also the reverse primer sequence is trimmed off and the trimmed mate pair reads are saved, otherwise both reads are discarded. Trimmed FastQ files are provided as input for the first step of alignment and variant calling.

Burrows-Wheeler transformation-based alignment is performed with BWA software v7.5a [35], and BAM files are obtained using samtools v1.19 [36] and Picard-tool v1.95 (http://broadinstitute.github.io/picard/).

GATK V3.1 is used for insertions/deletions realignment (with RealignTargetCreator, IndelRealigner and BaseRecalibrator) and variant calling (with UnifiedGenotyper) according to GATK Best Practices recommendations [37, 38].

A second round of alignment and variant calling is then applied, with the aim of individuating those mutations present on the same allele affected by allele drop-out, downstream of the primer sequence and covered by another amplicon (Fig. 1b). In this second round, reads generated from primers containing a mutation (both on the forward or the reverse sequence) are discarded (Fig. 1d) and the remaining reads are provided as input to the alignment and variant calling pipeline described above. The newly identified variants are merged with the ones obtained in the previous step to provide the final set of identified variants. The reads removal step has been implemented via an ad-hoc developed Perl script (*ReadsRemoval.pl*).

#### Coverage evaluation

Coverage evaluation was performed with GATK DepthOfCov and via an ad-hoc developed Perl script to find adjacent regions with average coverage (in terms of number of aligned reads) less than 30x. This threshold has been established according to [39]. The determination of uncovered or low-coverage regions in NGS applications is required when a complete sample sequencing is desired. Uncovered regions can be sequenced via other sequencing techniques (e.g.: Sanger sequencing or more accurate NGS techniques).

#### Variant annotation

Variant annotation was performed via Annovar software (table_annovar.pl, [40]). Mutations were considered pathogenic if they were absent from controls (i.e., dbSNP, and 1000 Genomes databases), predicted to alter the sequence of the encoded protein (nonsynonymous, nonsense, splice-site, frameshift, and insertion/deletion mutations) and to adversely affect protein function, with the use of *in silico* prediction software (SIFT, PolyPhen, LRT, MutationTaster and MutationAssessor).

Sanger sequencing was used for variant validation in the target genes and to cover all non-covered regions.

#### Amplivar pipeline

AmpliVar was downloaded from https://github.com/alhsu/AmpliVar and installed following the instructions. The hg19 version of the human genome in 2bit format (hg19.2bit) for Blat gfServer configuration was downloaded from the University of California, Santa Cruz online repository (https://genome.ucsc.edu/).

### Synthetic dataset generation

A synthetic dataset (called SD1) was created to simulate the configuration shown in Fig. 1b, where two single point mutations are present on the same allele, the first falling on a primer-matching region and the second downstream and covered by another amplicon.

First, a real dataset with a mutation on a primer-pairing region was identified. The region of interest was covered by two overlapping amplicons, here called A and B, as shown (see Additional file 1: Figure S1A). Reads generated from these amplicons were isolated and, as expected, only reads originated by A, whose primers matched a non-mutated region, contained the mutation with a percentage of about 50 %. Amplicon B, originated by primers pairing a mutated sequence, was affected by ADO and all the reads were obtained by the non-mutated allele, so that less than 1 % contained the mutation (see Additional file 1: Figure S1B). Following this procedure, FastQ files containing 3186 reads from amplicon A and 5484 reads from amplicon B were constructed.

Synthetic datasets were *in silico* constructed via Matlab R2015a software (Mathworks, Natick, MA). In all the reads generated by amplicon A and containing a mutation, a second mutation falling 5 bps downstream (not falling on primer pairing region and covered both by A and B amplicons) was introduced with a probability of 90 % (from not reported experiments, no significant difference is observed varying this percentage between 70 % and 100 %). In order to simulate the unbalanced amplifications of A and B (observed also in real experimental data, where the ratio between A and B reads was almost 37:63), synthetic datasets were constructed by randomly combining read pairs from both amplicons in different proportions from 0 % to 100 %, as described in Additional file 1: Table S1.

Similarly, a second synthetic dataset (called SD2) was constructed to simulate the presence of a single nucleotide insertion in the primer matching region. This dataset was identical to SD1 in terms of mutation percentage and amplicon composition (as described, see Additional file 1: Table S1), with the only exception that the single nucleotide mismatch in the primer matching region was replaced by a single nucleotide insertion.

All datasets were analyzed with both MiSeq and trimming pipelines.

## Results and discussion

### Coverage evaluation

A total of 119 samples were sequenced with TruSeq Custom Amplicon kit on MiSeq sequencer.

The average number of reads per sample varied from 385,919.3 [340,659.4÷431,180.4] for samples belonging to ATP1A2 panel to 499,105.6 [439,199÷559,012.2] for COL4. No correlation was observed between the total number of reads generated per sample and the panel dimension (R2 < <0.1, see Additional file 1: Figure S2A and Table S2 for details), nor with the number of samples loaded on the flow-cell per run (data not shown).

Coverage was evaluated as defined in Methods section and the percentage of not covered base pairs varied from 3.4 % (for COL4 panel) to 9.2 % (for Parkinson panel), showing an increasing trend with panel dimension (R2 = 0.5222, see Additional file 1: Figure S2B and Table S2 for details).

All samples had a sufficient number of reads (so that the average coverage per base was always greater than 500x). A negative correlation was found between average coverage (varying from 1398 for Parkinson panel to 9418 for ATP1A2 panel) and panel dimension, probably due to the unvaried average number of reads for all samples (R2 = 0.86, see Additional file 1: Figure S2C and Table S2 for details).

### Variant identification with trimming and MiSeq pipelines

Variant calling step was performed with both MiSeq and trimming pipelines.

**Fig. 3:**
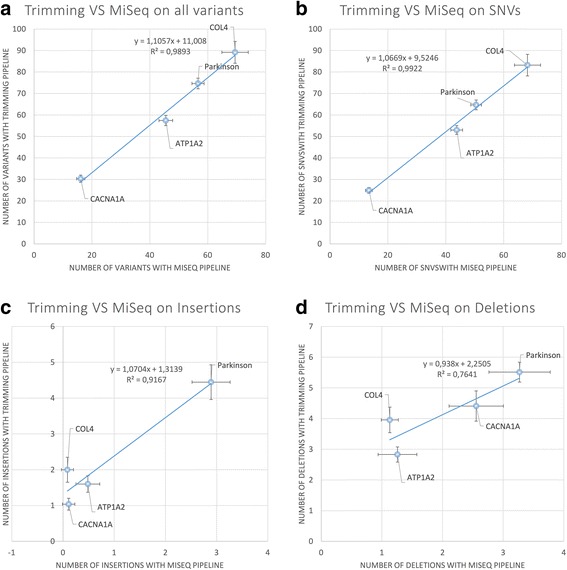
Comparison between trimming and MiSeq pipelines in terms of number of identified variants. All variants are shown in panel **a**, while only single nucleotide variants, insertions and deletions are shown in panel **b**, **c** and **d**, respectively. Dots represent the average on samples belonging to the same panel; error bars represent the 95 % confidence intervals. Solid line represents the linear regression fitting and equation and R2 are displayed in the plot

MiSeq pipeline identified an average number of variants per sample per panel ranging from 16.1 [14.7-17.5] for CACNA1A panel to 69.4 [64.9-74] for COL4 panel (see Fig. 3a and Additional file 1: Table S3 for details), while trimming pipeline identified a systematically higher number of variants (from 30.3 [28.7-32] to 89.2 [84.1-94.3], respectively, see Fig. 3 and Additional file 1: Table S3). The total number of variants identified with MiSeq and trimming pipelines was correlated (R2 = 0,98, see Fig. 3a) and the systematically higher number identified with trimming pipeline could be explained by less stringent filtration criteria on variant quality. Most of these variants were single nucleotide variations and less than 18 % were small insertions or deletions (see Additional file 1: Table S3). The correlations for single nucleotide variants, insertions and deletions are shown in Fig. 3b, c and d, respectively. The 99.5 % of MiSeq variants was also detected by trimming pipeline.

Interestingly, about 14 % of variants per sample fall on a primer-pairing region, thus highlighting the high impact of ADO-related artifacts in presence of one single nucleotide variation (see Additional file 1: Table S3). This percentage is not negligible and emphasizes that bioinformatics approaches, together with improvement and optimization of capturing kits, are indispensable to reduce artifacts.

The new discovery rate of trimming pipeline, expressed as percentage of mutations identified from trimming pipeline not found with MiSeq pipeline, is 64,8 % [64,8 %-70,8 %] for insertions and deletions and 26,6 % [24 %-29,3 %] for SNVs.

**Fig. 4:**
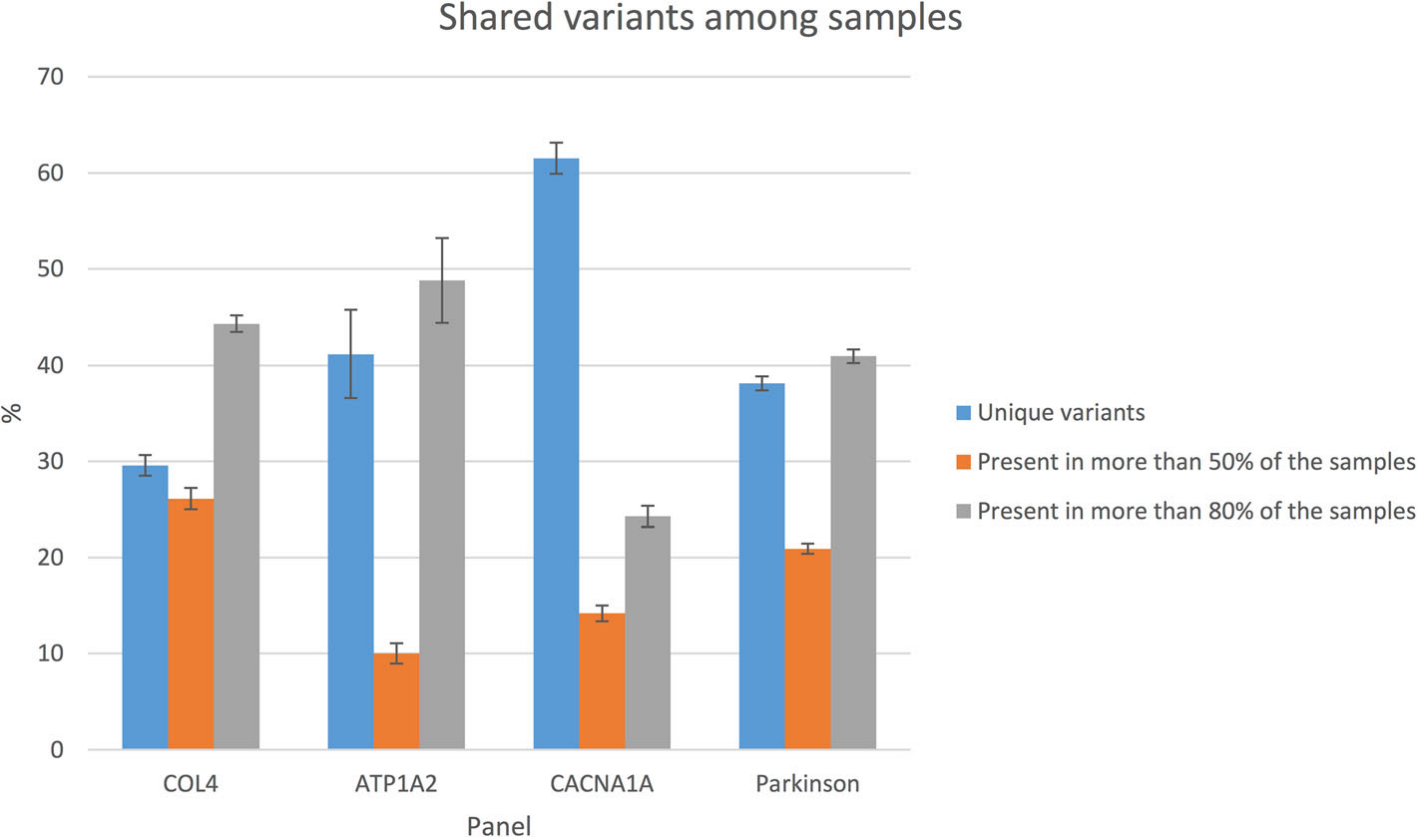
Percentage of shared variables between samples belonging to the same gene panel. In blue, the variants present in less than 50 % of the sample, in orange variants present in more than 50 % and less than 80 % of the samples and in grey variants present in more than 80 % of samples

Samples belonging to the same panel share a large number of mutations. In Fig. 4, variants present in at least 80 % of samples of the panel are shown in grey and in 50 % of the panel in orange, while the percentage of variants present in less than 50 % of samples (and considered as unique) is represented in blue. This phenomenon is common in both analytical methods.

In order to explore the nature of these shared mutations, we randomly selected ten of these variants that were sequence-verified via Sanger sequencing for all the samples of the panel (three from COL4 panel, three from CACNA1A, and three from ATP1A2 and one for Parkinson). All of them showed to be false positives, probably due to sequencing artifacts. Considering these shared variants as artifacts, the percentage of unique candidate variants ranges from 29,6 % [28.5 %-30.6 %] for COL4 panel to 61.5 % [59.2 %-63.1 %] for CACNA1A panel.

This finding suggests that highly shared variants may be candidate to be false positive, although these results are not conclusive and further investigations would be required to reveal the nature of such artifacts.

The number of predicted pathogenic variants in each cohort of patients varied between 0 (for ATP panel) and 36 for Parkinson panel (see Additional file 1: Table S4).

### Identification of a novel damaging-predicted variant for CACNA1A gene

**Fig. 5:**
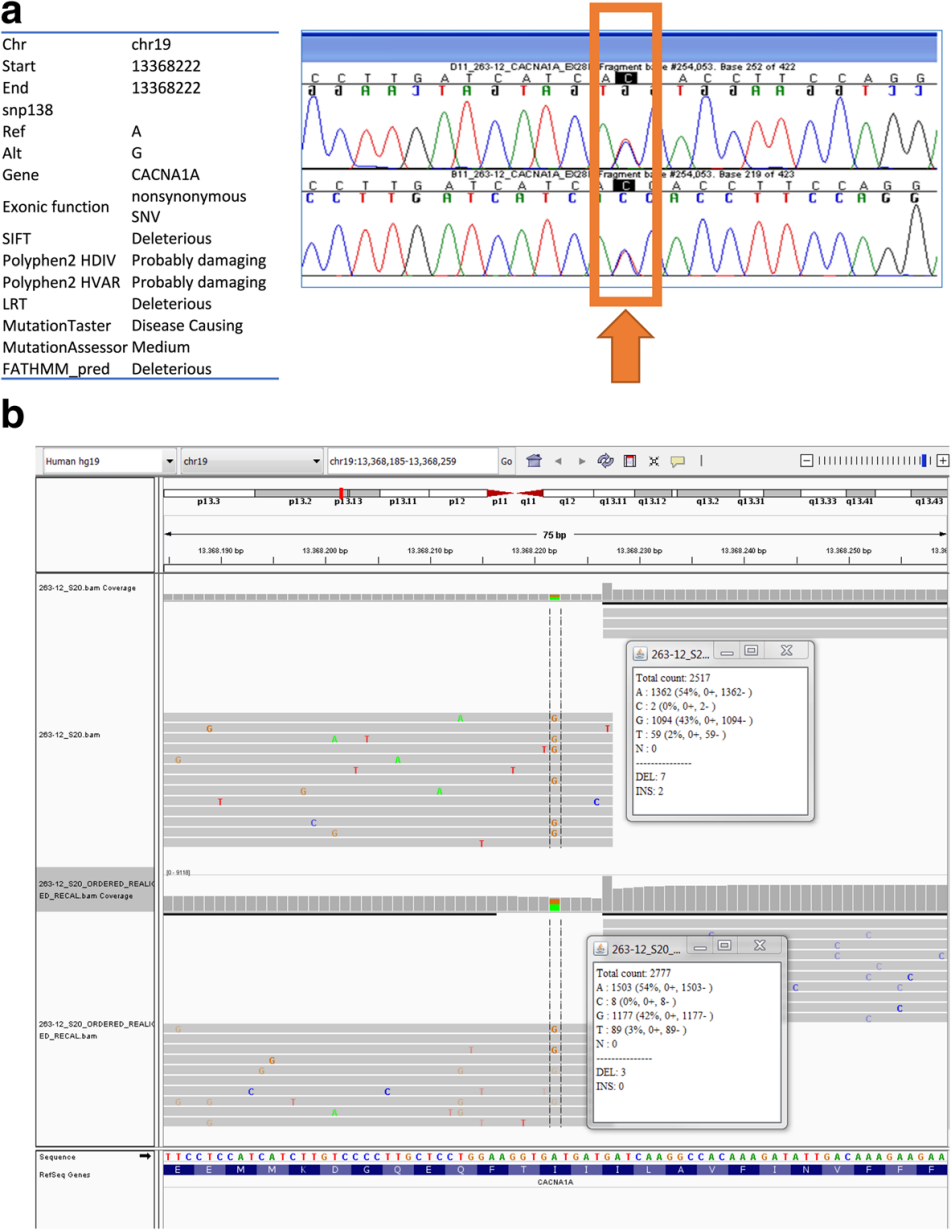
Newly identified CACNA1A mutation. **a** The newly identified heterozygous CACNA1A mutation NM_001127221:c.T4535C:p.I1512T was confirmed via Sanger sequencing on both DNA strands. **b** The mutation was correctly identified by both trimming and MiSeq pipelines. The first alignment results from MiSeq pipeline, while the second from trimming pipeline. Alignment qualities and parameters are highly similar between the different pipelines

A novel predicted damaging mutation on CACNA1A gene (NM_001127221:c.T4535C:p.I1512T) was present in one of CACNA1A samples and was correctly identified with both analytical procedures and Sanger sequence confirmed (see Fig. 5a). This mutation falls on a primer-pairing region and is covered by an additional amplicon. This configuration is shown (see Additional file 1: Figure S1A). As expected, reads generated from the primer pair that matches the mutated sequence (amplicon B) do not contain the mutation in the specified position, being identical to the reference for 99 %, while reads generated from the other overlapping amplicon (amplicon A) contain the mutation for 44 %. Relative abundances of reads from amplicon A and B are 5484 and 3186, respectively.

The alignment obtained with MiSeq and trimming pipelines are shown in Fig. 5b. In case the alignment is performed without the trimming step, the mutation is present in less than 20 % of reads and it is not detected during variant calling.

### Comparison of MiSeq and trimming pipelines performances on synthetic data

In order to evaluate the performances of MiSeq and trimming pipelines on a more complex configuration, not found in experimental data, an *in silico* evaluation procedure has been carried out.

Synthetic datasets have been *in silico* constructed as described in Methods section to reproduce the configuration where two single point mutations or an insertion and a single point mutation occur (see Fig. 1b).

In Table 2, the number of identified variants as a function of the percentage of reads belonging to amplicon A is shown. While trimming pipeline always identified the second mutation, the MiSeq pipeline could identify it in SD1 only if the percentage of reads coming from amplicon A was above 50 %, thus showing a threshold effect. Furthermore, MiSeq pipeline has similar performances to the first round of variant calling of trimming pipeline, while the second step is required to correctly determine the mutation. MiSeq performances improved for SD2, being able to detect the second mutation also if present at lower percentage.

**Table 2:** Pipeline performance evaluation on a synthetic dataset containing two mutations

% of reads from amplicon A	SD1			SD2		
	Trimming pipeline		MiSeq pipeline	Trimming pipeline		MiSeq pipeline
	First step of variant calling	Second step of variant calling		First step of variant calling	Second step of variant calling	
36.75	1	2	1	1	1	1
100	2	2	2	2	2	2
90	2	2	2	2	2	2
80	2	2	2	2	2	2
70	2	2	2	2	2	2
60	2	2	2	2	2	2
50	1	2	2	1	2	2
40	1	2	1	1	2	2
30	1	2	1	1	2	2
20	1	2	1	1	2	1
10	1	2	1	1	2	1
0	0	0	0	0	0	0

Results for both SD1 (two single nucleotide mutations) and SD2 (a single nucleotide insertion and a single nucleotide mutation) synthetic datasets are reported. The number of mutations found with trimming pipeline (during the first and second variant calling step) is reported. MiSeq pipeline performances for SD1 are comparable with the first step of variant calling of trimming pipeline and can identify the second mutation only if the percentage of reads from amplicon A (not affected by ADO) is above 50 %. For lower percentages, only trimming pipeline with the second step of variant calling can correctly identify the second mutation, even if amplicon A reads percentage lowers to 10 %. In SD2, trimming pipeline performances are identical to SD1, while MiSeq performances slightly improve, being able to identify the second mutation in two additional configurations (30 % and 40 %)

Sample 1 of the synthetic dataset SD1 containing two single point mutations (Table 2 and Additional file 1: Table S1) was also analyzed with AmpliVar tool [13] and it provided identical results than MiSeq pipeline, being able to determine only the first variant, falling on primer matching region. It should be noted that AmpliVar is not designed to manage complex configurations, as the ones reported in this work, and issues in variant calling, if variants overlap primer region, are a known limitation of the tool, due to the lack of primer soft-clipping [13].

## Conclusions

Amplicon-based NGS techniques are gaining great importance in the field of molecular-based diagnosis and research. Based on the targeted amplification of small portions of the genome, via sequence-specific probes, they suffer from the typical problems of PCR-based approaches, like nucleotide misincorporation, chimera formation and ADO [2, 7].

In this work, we focused on ADO-related artifacts and developed a bioinformatic methodology to manage such issue, in order to maximize the retrieved information from available sequencing data.

Our findings suggest that about 14 % of the mutations per sample, identified via customized Illumina panels, is potentially affected by this issue, since they fall on a primer matching sequence.

Different approaches have been proposed to address such problems, based on the definition and standardization of PCR protocols [7, 11], on specific bioinformatic pipelines for the analysis of such data and on the development of ad-hoc tools [13–15].

Although the presence of a single heterozygous mutation in a primer pairing sequence can be managed via primer sequence trimming, in presence of at least one additional amplicon covering the problematic region, more complex situations are not managed by these approaches.

Issues related to the presence of a second mutation (e.g., a causative mutation occurring on the same allele of a polymorphism falling on a primer pairing region) have been addressed by Chong et al. [BRCAPlus] by modifying the structure of the designed gene panel; while in a standard design one or two amplicons cover the region of interest, Chong et al. designed custom primers to obtain overlapping, redundant amplicons to over-cover target regions. Although this approach effectively manages such artifacts, more complex and expensive customized designs are required, thus imposing a trade-off between panel dimension and costs.

Our work allows increasing the amount of information that can be retrieved from NGS data obtained with amplicon panels without modifying probe design and, for this reason, it cannot overcome intrinsic panel limitations (e.g., allelic drop-out on regions covered by one only amplicon, the presence of a second mutation not covered by an additional amplicon). A trimming pipeline has been developed, based on two subsequent cycles of alignment and variant calling and has been compared to the proprietary Custom Amplicon workflow implemented on Illumina sequencer (Miseq pipeline).

We sequenced and analyzed 119 samples with four different newly designed Illumina TruSeq Custom Amplicon gene panels for neurological diseases. The percentage of not covered regions (with a non-coverage threshold of 30x) varies from 3.4 % for COL4 samples up to 9.2 % for Parkinson samples. Non-covered regions should be targeted via other methods (e.g.: Sanger sequencing, more specific gene panels, other sequencing techniques), thus constituting an additional effort to be accounted for to obtain the complete sequencing of the desired regions.

The performances of trimming and MiSeq pipelines have been compared in terms of number of identified variants and new discovery rate. Similar performances in the identification of a new predicted damaging single point heterozygous mutations on CACNA1A gene (NM_001127221:c.T4535C:p.I1512T), affected by ADO artifacts have been observed. The percentage of MiSeq mutations also identified via trimming pipeline is 99.5 %, thus suggesting that no loss of information occurs when using trimming pipeline compared to MiSeq pipeline.

Trimming pipeline has also been validated *in silico* on synthetic datasets, where a second mutation was introduced. Here, trimming pipeline outperformed MiSeq pipeline, correctly identifying the mutation even if the percentage of ADO-affected amplicons covering the region of interest rises up to 90 %. Here a threshold effect arises with MiSeq pipeline, being able to detect the second mutation if ADO-affected amplicons percentage is below 50 %.

Although the here described configuration has not been encountered in the analysis of our data and is probably not frequent, it is mandatory to adopt analytical procedures to manage it, particularly in diagnostics applications to avoid the clinical reporting of false negatives.

Although implemented for a specific platform (Illumina) this method is suitable for all amplicon-based applications and can be used both for paired-end and single-end reads.

Perl scripts for trimming pipeline implementation on TruSeq Custom Amplicon data are available for download at http://lab-bioinfo.unipv.it/index.php/it/dload.

## Additional file

Additional file 1: Supplementary figures and tables. (PDF 322 kb)

## Abbreviations

ADO: Allele drop-out
BAM: Binary-sequence alignment format
DAL: Diluted amplicon library
DLSO: Downstream locus specific oligo
DNA: Deoxyribonucleic acid
NGS: Next generation sequencing
PAL: Pooled amplicon library
PCR: Polymerase chain reaction
SAM: Sequence alignment format
ULSO: Upstream locus specific oligo
VCF: Variant calling format
