# Supplementary material for: Analysis of amplicon-based NGS data from neurological disease gene panels: a new method for allele drop-out management
Source: BMC Bioinformatics. 2016 Nov 8;17(Suppl 12):15–26. doi: 10.1186/s12859-016-1189-0 (PMC5123238; doi:10.1186/s12859-016-1189-0)
Supplement: Additional file 1: — Supplementary figures and tables. (PDF 322 kb) [file 12859_2016_1189_MOESM1_ESM.pdf]

Supplementary Figures

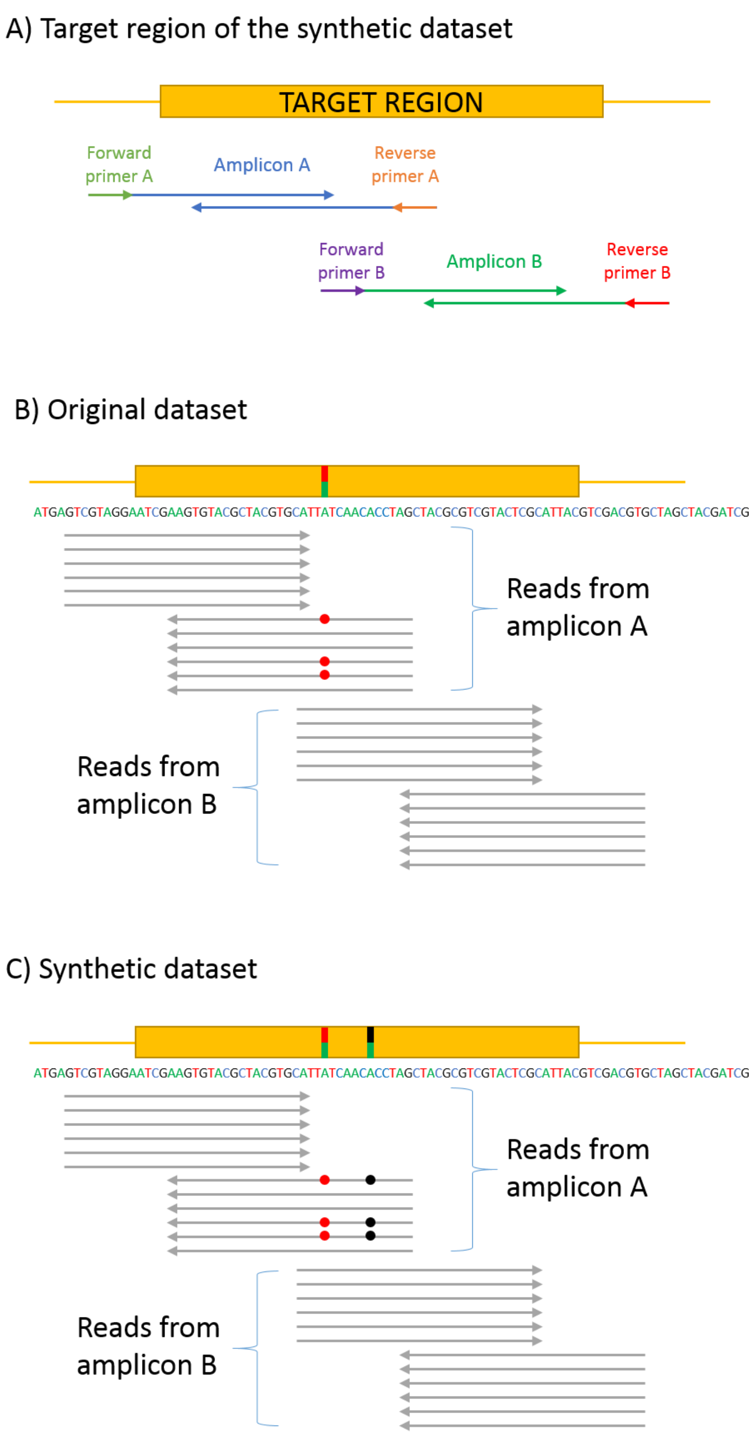

Figure S1 – Generation of a synthetic dataset. A) A target region is covered by two amplicons, A and B, originated by forward and reverse primers. B) A real dataset containing a heterozygous single nucleotide variant falling on a primer pairing region is here represented. Only reads generated from amplicon A, whose primers do not pair the mutated region, contain the variant at about 50% (red dots). C) A second mutation (black dots) is introduced to simulate the complex configuration when two mutations are present on the same allele, the first in a primer pairing region and the second downstream, covered by an additional amplicon. A second synthetic dataset containing a single nucleotide insertion instead of the single nucleotide mismatch (red dot) in the primer matching region has also been simulated.

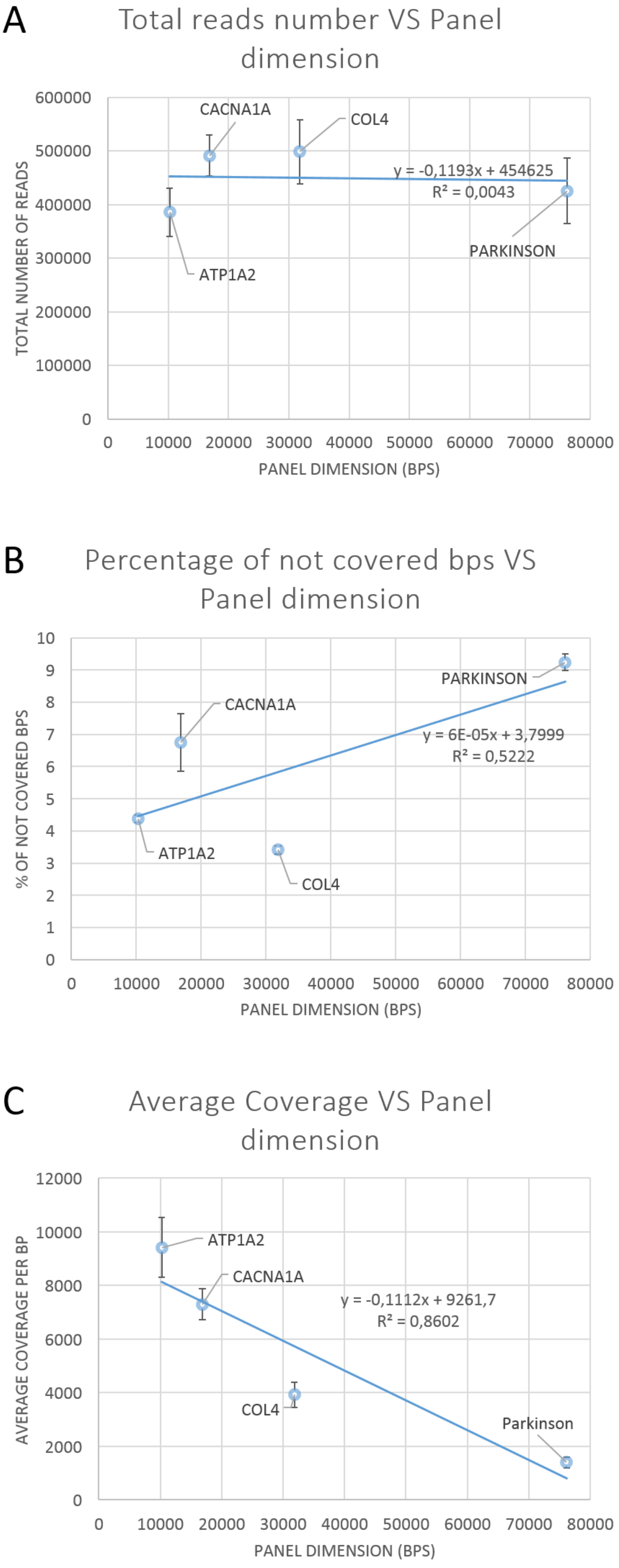

Figure S2 – A) Total number of reads per sample per panel as a function of panel dimension. B) Percentage of not covered base pairs per sample per panel, as a function of panel dimension. C) Average coverage per sample per panel, as a function of panel dimension. Dots represent the average on samples belonging to the same gene panel; error bars represent the 95% confidence intervals. Solid line represents the linear regression fitting and equation and R2 are displayed in the plot.

Supplementary Tables

Synthetic dataset	READS FROM AMPLICON A	READS FROM AMPLICON B	TOTAL READS	% of reads from amplicon A
1	3,186	5,484	8,670	36.75
2	3,000	0	3,000	100
3	2,700	300	3,000	90
4	2,400	600	3,000	80
5	2,100	900	3,000	70
6	1,800	1,200	3,000	60
7	1,500	1,500	3,000	50
8	1,200	1,800	3,000	40
9	900	2,100	3,000	30
10	600	2,400	3,000	20
11	300	2,700	3,000	10
12	0	3,000	3,000	0

Table S1 Synthetic datasets SD1 and SD2 have been generated in order to simulate the configuration of two single point mutations or of a single nucleotide insertion and a single nucleotide mutation, respectively, on the same allele. Here, the number of reads belonging to each amplicon is reported. Amplicon A contains both the mutations and simulates a situation of heterozygous state, while amplicon B, affected by ADO-related artefacts, contains reads generated from the non-mutated allele only. For each dataset (from 1 to 12), the composition is specified and the percentage of reads belonging to amplicon A is reported.

	Reads number	Average coverage (x)	Not covered regions (bps)	Not covered region (%)
Parkinson	425,826.8 [364,566.6÷487,087.2]	1,398 [1,197÷1,599]	7,035 [6,835÷7,235]	9.2 [9.0÷9.5]
COL4	499,105.6 [439,199÷559,012.2]	3919 [3,448÷4,389]	1,086 [1,046÷1,126]	3.4 [3.3÷3.5]
CACNA1A	491,537.8 [453,351÷529,724.6]	7,299 [6,732÷7,866]	1,137 [987÷1,287]	6.8 [5.9÷7.6]
ATP1A2	385,919.3 [340,659.4÷431,180.4]	9,418 [8,314÷10,523]	448 [437÷460]	4.4 [4.3÷4.5]

Table S2 – The statistics about the average number of reads per sample for each panel, the average coverage in terms of aligned reads per base and the extension of the non-covered regions are here reported. Numbers are the average on the samples belonging to the panel and 95% confidence interval is reported between brackets.

	MiSeq pipeline			Trimming pipeline			Number of variants falling on a primer pairing region
	**SNVs**	**In**	**Del**	**SNVs**	**In**	**Del**	
Parkinson	50.5 [48.6÷52.3]	2.9 [2.5÷3.3]	3.3 [2.8÷3.8]	64.7 [62.4÷67]	4.4 [4÷4.9]	5.5 [5.2÷5.8]	13.6 [12.8÷14.5]
COL4	68.2 [63.7÷72.7]	0.1 [0÷0.2]	1.1 [1÷1.3]	83.2 [78.2÷88.3]	2 [1.7÷2.3]	4 [3.5÷4.4]	11.6 [10.7÷12.4]
CACNA1A	43.8 [41.7÷45.8]	0.1 [0÷0.2]	2.6 [2.1÷3]	24.9 [23.5÷26.3]	1 [0.9÷1.2]	4.4 [3.9÷4.9]	3.1 [2.6÷3.7]
ATP1A2	13.4 [12.3÷14.6]	0.5 [0.3÷0.7]	1.3 [0.9÷146]	53 [50.9÷55.1]	1.6 [1.8÷1.4]	2.8 [2.6÷3.1]	7.7 [7.1÷8.4]

Table S3 - Number of variants identified with the two different pipelines, grouped in Single Nucleotide Variants, Insertions and Deletions. The number of variants falling on a primer (identified by trimming pipeline) is also reported to highlight the significance of ADO-related artefacts. Numbers are the average on the samples belonging to the panel and 95% confidence interval is reported between brackets.

	Number of predicted pathogenic mutations in the full cohort of patients for each panel
Parkinson	36
COL4	5
CACNA1A	14
ATP1A2	0

Table S4 - Number of distinct predicted pathogenic mutations in the full cohort of patients for each tested panel. Mutations were considered predicted pathogenic when requirements reported in the *Variant annotation* subsection (main text, Methods, Bioinformatic data analysis) were met.
